# Integrated transcriptomic and functional analyses reveal that NOX2 inhibition rewires the inflammatory landscape of macrophages

**DOI:** 10.3389/fimmu.2026.1731888

**Published:** 2026-02-18

**Authors:** Iswarya Muthukumarasamy, Sharleen M. Buel, Jennifer M. Hurley, Jonathan S. Dordick

**Affiliations:** 1 Department of Chemical and Biological Engineering, Rensselaer Polytechnic Institute, Troy, NY, United States; 2 Center for Biotechnology and Interdisciplinary Studies, Rensselaer Polytechnic Institute, Troy, NY, United States; 3 Department of Biological Sciences, Rensselaer Polytechnic Institute, Troy, NY, United States; 4 Rensselaer-Mount Sinai Center for Engineering and Precision Medicine, New York, NY, United States

**Keywords:** GSK2795039, mouse macrophages, NADPH oxidase 2, NOX2 inhibition, RNA sequencing, ROS, transcriptomics

## Abstract

**Background:**

Macrophages are dynamic immune cells whose phenotype and function are shaped by environmental cues, including inflammatory stimuli and oxidative stress. A major source of macrophage-derived reactive oxygen species (ROS) is NADPH Oxidase 2 (NOX2), which is critical for microbial defense but also contributes to redox signaling and inflammatory responses. This increase in NOX2-based ROS can be both beneficial and detrimental, leading to the desire to modulate this key inflammatory pathway pharmacologically. However, while NOX2-driven ROS are well studied in host defense, the underlying macrophage transcriptional reprogramming that leads to inflammatory phenotypes, and the changes that occur to this programming under pharmacological inhibition, remain unclear.

**Methods:**

To address this gap, we used the selective small-molecule inhibitor GSK2795039 (GSK) to acutely block NOX2 activity in primary bone marrow–derived macrophages (BMDMs) under basal and lipopolysaccharide (LPS)-stimulated conditions. RNA sequencing and functional assays were performed to uncover the role of inflammation mediation due to NOX2 on transcriptional changes in macrophages.

**Results:**

RNA sequencing revealed that GSK alone induced modest transcriptional changes in resting macrophages, largely restricted to metabolic and stress-associated pathways. In contrast, co-treatment with LPS and GSK markedly reprogrammed the macrophage transcriptome, attenuating classical pro-inflammatory responses while enriching pathways associated with anti-inflammatory activation, tissue repair, extracellular matrix remodeling, and oxidative phosphorylation. Functional assays validated these transcriptomic findings. NOX2 inhibition under LPS activation reduced both intracellular and extracellular ROS, suppressed pro-inflammatory cytokine secretion (TNF-α, IL-6, IL-1β), and enhanced anti-inflammatory cytokines (IL-4, IL-10).

**Conclusion:**

Together, these results demonstrate that NOX2 inhibition does not broadly reprogram macrophages in the resting state but reshapes the inflammatory landscape of LPS-stimulated pro-inflammatory macrophages, shifting them toward a reparative, anti-inflammatory state even in the presence of strong activating stimuli. Our findings provide mechanistic insight into the immunomodulatory potential of NOX2 inhibition in inflammatory models.

## 1 Introduction

Macrophages are highly plastic immune cells central to both host defense and the resolution of inflammation (1). They react dynamically to environmental cues by adopting distinct phenotypes that control various aspects of the immune response (1, 2). Under conditions of infection or tissue damage, macrophages can polarize into either a pro-inflammatory or an anti-inflammatory dominant state, a process crucial for balancing pathogen defense with tissue repair (1, 3, 4). The pro-inflammatory dominant state, often triggered by pathogen-associated molecular patterns (PAMPs) such as lipopolysaccharide (LPS), drives macrophages toward a phenotype characterized by the secretion of cytokines (e.g., TNF-α, IL-6, IL-1β), chemokines, and the production of reactive oxygen species (ROS) (5). LPS-mediated activation is primarily facilitated through the Toll-like receptor 4 (TLR4) signaling axis, which stimulates downstream NF-κB and MAPK pathways to promote expression of inflammatory mediators (5, 6).

One of the key downstream effectors of TLR4 activation in macrophages is NADPH oxidase 2 (NOX2), a multi-subunit enzyme complex responsible for generating superoxide and other ROS during the respiratory burst (7, 8). In its inactivated state, NOX2 comprises a set of cytoplasmic and transmembrane sub-units (7–9). Activation of NOX2 happens through a complex series of protein/protein interactions and translocations resulting in the combination of the cytosolic and transmembrane units creating a full functional enzyme complex, which then produces superoxide by transferring an electron from NADPH (present in the cytosol) to an oxygen molecule (present in the extracellular space) (8, 9). While ROS play important roles in microbial killing and intracellular signaling, excessive ROS production can propagate inflammatory damage and contribute to chronic inflammatory diseases (9, 10). In this context, pharmacological inhibition of NOX2 has been proposed as a strategy to limit inflammation and oxidative stress. GSK2795039 (GSK), a small-molecule NOX2 inhibitor that competes for the attachment site of NADPH on the enzyme complex, has been shown to attenuate ROS production and modulate inflammatory outcomes in various disease models, including cardiovascular injury, neuroinflammation, and fibrosis (11–13). While much is known about how NOX2 is stimulated and repressed by these small molecule inhibitors, little is known about how NOX2 inhibition reprograms macrophage gene expression and function, leaving a critical gap in our understanding of its downstream impact. Clarifying how NOX2 inhibition shapes macrophage responses is therefore essential for evaluating its therapeutic potential in inflammatory disease contexts.

Accordingly, we sought to investigate the extent to which NOX2 inhibition via small molecules reshapes the macrophage transcriptome and functional phenotype in order to better understand its role in regulating inflammation. In this study, we investigated the transcriptomic consequences of NOX2 inhibition in primary bone marrow-derived macrophages (BMDMs) using GSK, both in basal conditions and under LPS-induced pro-inflammatory activation. Transcriptomic profiling of BMDMs revealed that lipopolysaccharide (LPS) stimulation induces a robust pro-inflammatory transcriptional program. In contrast, treatment with the NOX2 inhibitor GSK, either alone or in combination with LPS, resulted in distinct transcriptional reprogramming. GSK treatment in naïve macrophages led to modest transcriptional changes, primarily enriched in metabolic pathways such as cholesterol efflux and amino acid transport. Notably, GSK co-treatment with LPS (LPS and GSK) attenuated the classical LPS-induced inflammatory signature and promoted expression of genes associated with alternative (anti-inflammatory) macrophage activation. Functional assays validated the transcriptomic findings: LPS and GSK treatment significantly reduced both intracellular and extracellular ROS production compared to LPS alone, and decreased secretion of pro-inflammatory cytokines while enhancing anti-inflammatory cytokines. These findings offer mechanistic insight into how the inhibition of NOX2-derived ROS shapes macrophage function, indicating that NOX2 inhibition counteracts LPS-mediated macrophage activation and promotes a metabolic and transcriptional shift toward a reparative, anti-inflammatory dominant phenotype.

## 2 Materials and methods

### 2.1 Bone marrow derived macrophage extraction and circadian synchronization

Bone marrow was harvested from the tibias and femurs of 3-month-old male Per2:Luc (C5BL/6) mice bred in-house from stocks maintained at Jackson Laboratory and were used for all experiments involving primary BMDMs. Mice were kept in standard housing on a strict lighting schedule of 12L:12D to maintain synchronized circadian rhythms and fed standard rodent chow *ad libitum*. They were euthanized using CO_2_ asphyxiation (50% flow rate of chamber volume per minute with CO_2_) followed by cervical dislocation to ensure non-recovery. The experiments conducted with mice were done in accordance with the guidelines set by the National Institutes of Health Office of Intramural Research and were approved and supervised by the Rensselaer Polytechnic Institute Animal Care and Use Committee. The bone marrow progenitor cells were plated in 6-well cell culture plates and 35 mm cell culture dishes and induced to differentiate into macrophages using DMEM (ThermoFischer Scientific, Waltham, MA) supplemented with M-CSF (ThermoFischer Scientific) and 10% FBS (Gibco, ThermoFischer Scientific), following established protocols (**Supplementary Figure S1a**) (12, 14). Given our prior work demonstrating that circadian oscillations influence the expression of key inflammatory mediators displaying a time-of-day effect, we employed the serum shock synchronization protocol to ensure all macrophage cultures were aligned to the same circadian phase at the time of treatment and sample collection (12, 14). To do so, after a 7-day differentiation period, the resulting macrophages were synchronized by subjecting them to serum starvation for 24 h in serum-free media, followed by a 2 h serum shock with 50% FBS (**Supplementary Figure S1a**) (12, 14–16). Post synchronization, the cells were switched back to standard growth media and allowed to recover from the serum shock for 16 h to attain homeostasis (12, 14). To validate circadian synchronization, cells grown in 35 mm cell culture dishes were sealed with grease and glass cover slips, and luminescence was continuously recorded for 5 days using the LumiCycle32 (Actimetrics, Wilmette, IL), with cells maintained in Leibovitz media (ThermoFischer Scientific) supplemented with Luciferin and 10% FBS (14). To obtain RNA for transcriptomic analysis, the synchronized macrophages from three biological replicate mice (n=3) were exposed to 25 µM GSK (Millipore Sigma, Burlington, MA), LPS (1 µg/mL) (Invitrogen, Waltham, MA) or LPS (1 µg/mL) + GSK (25 µM) for 24 h starting at HPS20 (20 h post-shock), with untreated samples acting as the control. HPS20 was chosen as an appropriate time point to allow adequate time for the cells to recover from the serum-shock to avoid artifactual gene expression that occurs immediately post serum-shock (**Supplementary Figure S1b**) (12, 14). GSK was dissolved in DMSO at 30 mM and diluted into cell culture media to a final concentration of 25 µM, corresponding to 0.083% (v/v) DMSO; to ensure consistency, all treatments, including controls (“Control” condition), were standardized with the vehicle (0.1% (v/v) DMSO).

### 2.2 RNA sequencing and data analysis

Cells collected for RNA sequencing were flash frozen and sent to Azenta for RNA library preparation, sequencing, and initial analysis (South Plainfield, NJ). RNA sequencing data were analyzed using R (version 4.3.3) in RStudio. Differentially expressed genes (DEGs) were identified using DeSeq2 (version 1.42.1). Genes with log2 fold change >1.5 and adjusted p-value < 0.05 were classified as significant DEGs and were used for gene set enrichment analysis (GSEA) and gene ontology (GO) analysis. GO analysis was performed using Metascape and ShinyGO Version 0.80, and GSEA was conducted using the software available on the GSEA-MSigDB portal (https://www.gsea-msigdb.org) the mouse MH: Hallmark (containing 50 gene sets) and M2: Curated (containing 2710 gene sets) collections from the molecular signature database (https://www.gsea-msigdb.org/gsea/msigdb/index.jsp).

### 2.3 ROS measurement and cell viability

ROS production in BMDMs in response to LPS (1 µg/mL) stimulation, with or without GSK (25 µM), was quantified using two distinct assays. Intracellular superoxide levels were assessed using the DCFDA/H2DCFDA Cellular ROS Assay Kit (Abcam, Cambridge, MA), while extracellular hydrogen peroxide production was measured with the Amplex Red Hydrogen Peroxide/Peroxidase Assay Kit (Invitrogen). In both assays, BMDMs were seeded in black-walled, flat-bottom 96-well plates at a density of 10^5^ cells/mL and allowed to reach confluence. At 90% confluency, cells were serum shock synchronized (as described above) and incubated with LPS with/without GSK for 2 h starting at HPS20 in assay media. Fluorescence was recorded using a SpectraMax plate reader (Molecular Devices, San Jose, CA). Post measurement, the media was aspirated and replaced with 90 µL of fresh media and 10 µL of PrestoBlue reagent (ThermoFischer Scientific). Cell viability was confirmed across treatment groups using the PrestoBlue cell viability assay to ensure these doses did not induce cytotoxicity (**Supplementary Figure S2**). Cells were further incubated at 37°C in a 5% CO_2_ atmosphere for 10 min. Fluorescence was subsequently measured at excitation/emission wavelengths of 560/590 nm, and cell numbers were determined by correlating fluorescence values to a standard curve. The fluorescence values from the ROS assays were normalized to cell numbers, determined via the Presto Blue Cell Viability Assay.

### 2.4 ELISA

Sandwich enzyme-linked immunosorbent assay (ELISA) was employed to quantify cytokine production in BMDMs. Cells were seeded in 96-well plates at a density of 10^5^ cells/mL and cultured until confluent. The cells were then serum shock synchronized (as described above) and incubated with LPS with/without GSK for 24 h starting at HPS20. Samples with no treatment were used as a control. After incubation, supernatants were collected and the concentrations of pro-inflammatory cytokines, TNF-α, IL-6 and IL-1β, and anti-inflammatory cytokines, IL-4 and IL-10, were quantified using the DuoSet ELISA Kit (R&D Systems, MN, USA). Data was analyzed using GraphPad Prism Version 10.

### 2.5 Statistical analysis

RNA Sequencing data were analyzed as described in the above section. For ELISA and ROS measurement data, one-way ANOVA was employed to analyze biological replicates, with each data point representing triplicate biological replicates, each further comprising triplicate technical replicates. All statistical analyses were conducted using GraphPad Prism Version 10 or Microsoft Excel.

## 3 Results

### 3.1 RNA-Seq analysis highlighted pro-inflammatory signatures and pathway enrichment in LPS-treated macrophages

**Figure 1:**
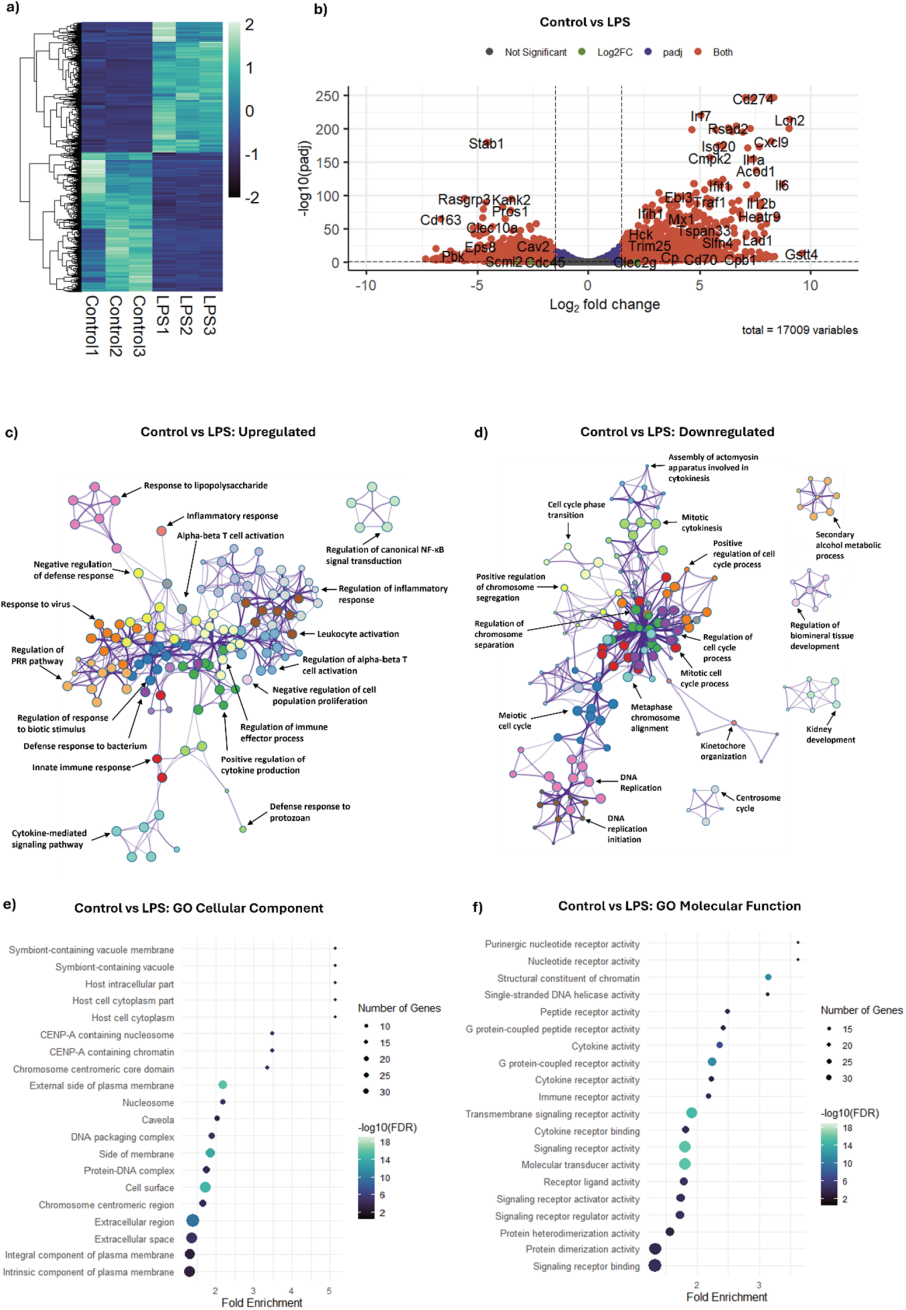
LPS activation drives widespread transcriptional reprogramming and pro-inflammatory gene expression in macrophages. Differential gene expression analysis was performed on RNA-seq data obtained from serum shock synchronized BMDMs exposed to LPS for 24 h starting at HPS20 and compared to control (untreated/inactivated) macrophages. **(a)** Heatmap with unsupervised hierarchical clustering showing the distribution of significant DEGs between control and LPS treatment (24 h). Each column represents one sample, and each row represents one DEG. Z-scores were calculated from normalized gene expression values and were plotted on a scale of -2 to 2. The z-scores are displayed in a dark blue-light green color scheme with dark blue and light green representing low and high expression, respectively. **(b)** Volcano plot indicating the significantly upregulated and downregulated DEGs via LPS treatment in comparison to control incubation (filter: |Log2 Fold Change| > 1.5 and p-adj < 0.05). Grey dots indicate genes that did not meet significance thresholds. Green dots indicate genes with a fold change above the cutoff that were not statistically significant (p-adj ≥ 0.05). Blue dots represent genes that were statistically significant (p-adj < 0.05) but with a fold change below the cutoff. Red dots represent genes that met both criteria (p-adj < 0.05 and |Log2 Fold Change| > 1.5) and thus were classified as significant DEGs. Representative DEGs are labeled. Gene ontology analysis based on GO Biological processes was performed using Metascape network analysis on the **(c)** upregulated and **(d)** downregulated DEGs via LPS treatment vs. control. Significant (p < 0.05) ontology terms were grouped based on similar memberships into color-coded clusters and represented as network plots, with an individual node representing an enriched term. Each color-coded cluster is represented by one representative term. Minimum overlap value was set as 3 enriched terms, and minimum enrichment was set as 1.5. **(e)** GO Cellular Component and **(f)** GO Molecular Function based enrichment analysis summarized for the top 20 enrichment terms. Categories were selected based on their False Detection Rate (FDR) values and sorted based on their Fold Enrichment, with the point sizes representing the number of genes in each enrichment term and the point color representing the -Log_10_(FDR) value. FDR cutoff was set as 0.05. The complete enrichment results for all three pathway analyses are provided in FASTQ files.

NOX2 plays a pivotal role in generating ROS and regulating various cellular processes, including immune responses, inflammation, and oxidative stress management in macrophages (17). Therefore, to understand the effects of the inhibition of NOX2 on the pro-inflammatory processes within a macrophage, we first needed to understand the molecular landscape of an inflamed macrophage. LPS is an established inducer of the pro-inflammatory immune response, facilitated primarily through the activation of Toll-like receptor 4 (TLR4) signaling (18). As a baseline, we investigated the exposure of BMDMs to LPS (1 µg/mL) for 24 h. The 1 µg/mL concentration of LPS was selected based on previous studies done in the field and our own previous work demonstrating its robust induction of pro-inflammatory signaling and ROS production in BMDMs (12, 19–23). To do so, transcriptional profiles of LPS and non-LPS (control)-treated cell cultures were compared using RNA-seq. Differential gene expression analysis was performed using DeSeq2, with the inactivated cells serving as control. Cutoffs of |Log_2_FoldChange| > 1.5 and a false discovery adjusted p-value < 0.05 were used. This approach identified 3,208 genes as significant differentially expressed genes (DEGs) in LPS-activated macrophages vs. the control, among the 17,040 analyzed genes. A heatmap of the 3,208 significant DEGs with hierarchical clustering indicated a distinct transcriptional response of macrophages after LPS stimulation (**Figure 1a**). A volcano plot was generated to highlight the upregulated and downregulated significant DEGs (**Figure 1b**). Among the 3,208 significant DEGs, 1,548 genes were upregulated (Log_2_FoldChange > 1.5 and a false discovery adjusted p-value < 0.05) and 1,660 genes were downregulated (Log_2_FoldChange < -1.5 and a false discovery adjusted p-value < 0.05) in LPS-activated macrophages in comparison to the control. Not surprisingly, activation of macrophages using LPS led to the expression of various pro-inflammatory genes, including cytokines, chemokines, and other mediators that play a key role in the inflammatory cellular phenotype (24). Among the significantly upregulated genes upon LPS activation, *Il6*, *Il1a*, *Il12b*, *Cd274*, *Cxcl9*, and *Lcn2* play important roles in mediating the macrophage inflammatory responses (**Figure 1b**) (25). Conversely, several genes were significantly downregulated upon incubation in the presence of LPS, including *Stab1*, *Cd163*, *Clec10a*, and *Pros1* (**Figure 1b**), reflecting the transition of macrophages into a pro-inflammatory activated state.

To gain deeper insight into the biological processes affected by LPS activation in mouse BMDMs, Gene Ontology (GO) pathway enrichment analysis was performed using the significantly upregulated and downregulated DEGs. Specifically, each DEG was mapped to its associated GO term related to either biological processes, molecular functions, or cellular components in which the DEG is involved. After mapping, genes from our experimental dataset were then compared to annotated gene sets in the GO database to highlight clusters of related functions or processes that are enriched as a result of LPS treatment in comparison to the untreated control. LPS activation resulted in increased representation of clusters related to response to lipopolysaccharide (GO:0032496, fold enrichment = 3.27), confirming LPS-mediated activation of the macrophages (**Figure 1c**). Clusters of ontology terms relating to immune and inflammatory responses, such as inflammatory response (GO:0006954, fold enrichment = 2.3), regulation of inflammatory response (GO:0050727, fold enrichment = 2.8), and positive regulation of cytokine production (GO:0001819, fold enrichment = 2.9), demonstrated increased representation in the upregulated DEGs, suggesting activation of pro-inflammatory pathways and the upregulation of genes involved in cytokine production (26, 27).

Among the downregulated DEGs, clusters related to cell cycle, including mitotic cell cycle process (GO:1903047, fold enrichment = 2.5), cell cycle process (GO:0010564, fold enrichment = 1.8), cell cycle phase transition (GO:0044770, fold enrichment = 2.3), positive regulation of cell cycle process (GO:0090068, fold enrichment = 2.4), and mitotic cytokinesis (GO:0000281, fold enrichment = 2.6) were significantly overrepresented (**Figure 1d**). The downregulation of these processes correlated with the known effect of LPS on macrophage cell proliferation and division (28, 29), which is further supported by a reduction in gene expression involved in DNA replication (GO:0006260, fold enrichment = 2.3), DNA replication initiation (GO:0006270, fold enrichment = 4.9), and metaphase chromosome alignment (GO:0051310, fold enrichment = 2.8).

Cellular component-based GO pathway enrichment analysis was used to further investigate the cellular localization and potential functional roles of the significant DEGs expressed in LPS-activated macrophages in comparison to the control (**Figure 1e**). The most significantly enriched terms were associated with the symbiont-containing vacuole (GO:0020003, fold enrichment = 5.14) and host cell components. Other enriched sets of terms, including those related to chromatin and nucleosome components, cell surface and membrane components, DNA packaging, extracellular region, and intrinsic components of plasma membrane, further emphasize the reorganization of genetic and cellular structures upon LPS activation (2, 30, 31).

Finally, molecular function based GO pathway enrichment analysis indicated LPS-mediated activation of gene expression related to receptor activities, including purine nucleotide receptor activity (GO:0001614, fold enrichment = 3.63), nucleotide receptor activity (GO:0016502, fold enrichment = 3.63), peptide receptor activity (GO:0001653, fold enrichment = 2.47), G protein-coupled peptide receptor activity (GO:0008528, fold enrichment = 2.42), cytokine receptor activity (GO:0004896, fold enrichment = 2.22), and immune receptor activity (GO:0140375, fold enrichment = 2.19), thus reflecting the role of various receptors in extracellular signaling and mediating macrophage activation and inflammatory response (**Figure 1f**) (26). Similar to observations made from biological process pathway analysis, the increased enrichment of cytokine activity (GO:0005125, fold enrichment = 2.35) along with cytokine receptor binding (GO:0005126, fold enrichment = 1.8) further support the importance of activated cytokines in LPS-activated BMDMs (27). Genes relating to signal transduction, including transmembrane signaling receptor activity (GO:0004888, fold enrichment = 1.9), signaling receptor activity (GO:0038023, fold enrichment = 1.8), signaling receptor activator activity (GO:0030546, fold enrichment = 1.73), signaling receptor regulator activity (GO:0030545, fold enrichment = 1.72), and signaling receptor binding (GO:0005102, fold enrichment = 1.32) were enriched, indicating the upregulation of genes associated with intracellular signaling cascades and resulting in the distinct characteristics of the pro-inflammatory phenotype (32). Overall, the addition of LPS resulted in a multifaceted activation of receptors, cytokines, chemokines, signaling pathways, and protein-protein interactions that transform the transcriptional profile in macrophages, aligning with what has been shown previously (1, 3, 33–36).

### 3.2 NOX2 inhibition by GSK reprogrammed the transcriptome in naïve macrophages to a stress response phenotype

Having replicated what was known about the landscape of an activated macrophage, we next sought to determine how the inhibition of NOX2 affected a macrophage prior to its activation. Inhibition of NOX2 can be affected via several methods. Of these methods, we chose the small-molecule inhibitor, GSK, rather than a genetic NOX2 knockout model because pharmacological inhibition provides acute and reversible suppression of NOX2 activity, avoids potential developmental compensation inherent to knockout systems, and more closely reflects a clinically relevant therapeutic strategy (37, 38). The inhibition of NOX2 via GSK has been linked to changes in metabolic activity, reductions in inflammation, and modulations to redox signaling, but little is known about the effects of GSK on a macrophage at the molecular level (11–13, 17) To first study the effect of GSK-mediated NOX2 inhibition on the molecular reprogramming of BMDMs independently of the activation of a macrophage, serum-shock synchronized cells were incubated with and without GSK (25 µM) for 24 h starting at HPS20. After 24 h of incubation, cell samples were collected, and RNA sequencing (DESeq2) was performed to elucidate the changes at the transcriptional level upon GSK-mediated NOX2 inhibition prior to macrophage activation. The 25 µM concentration of GSK was selected based on prior studies demonstrating effective NOX2 inhibition in primary immune cells under inflammatory conditions (12, 23, 39–42).

**Figure 2:**
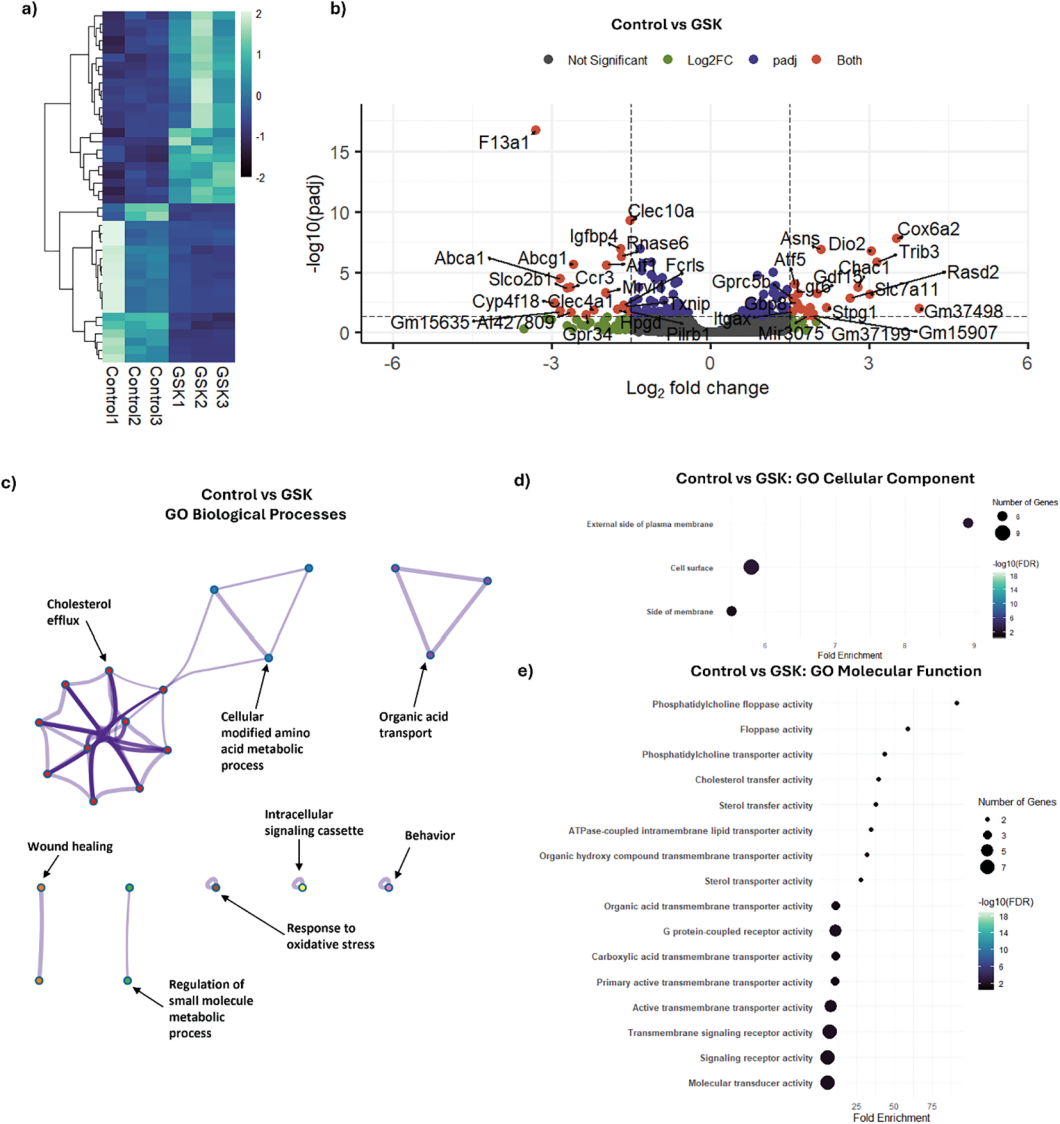
Pharmacological NOX2 inhibition with GSK induces transcriptomic changes related to stress response pathways. DeSeq2-based differential gene expression analysis was performed on RNA-Seq data obtained from serum shock synchronized BMDMs exposed to GSK for 24 h starting from HPS20 and compared to control. **(a)** Heatmap with unsupervised hierarchical clustering showing the distribution of significant DEGs following GSK treatment vs. control. Z-scores were calculated from normalized gene expression values and were plotted on a scale of -2 to 2, with each column representing one sample and each row representing one DEG. The z-scores are displayed in a dark blue-light green color scheme with dark blue and light green representing low and high gene expression, respectively. **(b)** Volcano plot indicating the significant DEGs (applied filter: |Log_2_FoldChange| >1.5 and p-adj < 0.05) from GSK treatment vs. control. Grey dots indicate genes that did not meet significance thresholds. Green dots indicate genes with a fold change above the cutoff that were not statistically significant (p-adj ≥ 0.05). Blue dots represent genes that were statistically significant (p-adj < 0.05) but with a fold change below the cutoff. Red dots represent genes that met both criteria (p-adj < 0.05 and |Log2 Fold Change| > 1.5) and thus were classified as significant DEGs. Representative DEGs are labeled. **(c)** Gene Ontology analysis based on GO Biological Processes was performed using Metascape on the entire set of significant DEGs following GSK treatment in comparison to control. Significant (p < 0.05) ontology terms were grouped based on similar memberships into color-coded clusters and are represented as network plots, with an individual node representing an enriched term. Each color-coded cluster is represented by one representative term. The minimum overlap value was set as 3 enriched terms, and minimum fold enrichment was set to 1.5. **(d)** GO Cellular Component, and **(e)** GO Molecular Function based Gene Ontology analysis of the significant DEGs indicating enrichment of pathways upon GSK treatment. Categories were selected based on their False Detection Rate (FDR) values and sorted based on their Fold Enrichment, with the point sizes representing the number of genes in each enrichment term and the point color representing the -Log_10_(FDR) value. FDR cutoff was set as 0.05. The complete enrichment results for all three pathway analyses are provided in FASTQ files.

Similar to LPS activation, DEGs that had a |Log_2_FoldChange| > 1.5 and an adjusted p-value < 0.05 in the GSK-exposed samples were considered significant. As a result of this filtering, 42 significant DEGs were identified in GSK-treated samples in comparison to the non-GSK-treated control samples from the pool of 17,763 analyzed genes. Unsupervised hierarchical clustering of all DEGs indicated distinct transcriptional patterns between GSK-treated samples and controls (**Figure 2a**). Of the 42 significant DEGs in the GSK-treated condition, 23 genes were upregulated (Log_2_FoldChange > 1.5 with a false discovery adjusted p-value < 0.05), and 19 genes were downregulated (Log_2_FoldChange < -1.5 and a false discovery adjusted p-value < 0.05) due to GSK treatment in comparison to the control (**Figure 2b**). *Cox6a2*, *Slc7a11*, *Trib3*, and *Dio2*, among others, showed upregulation in the presence of GSK compared to the control. Conversely, *F13a1*, *Abca1*, *Abcg1*, *Clec4a*, *Ccr3*, and *Slco2b1*, among others, showed downregulation in comparison to the control. Together, these genes suggest a modest shift in the macrophage towards altered metabolic and stress-adaptive states, with accompanying dampening of certain immune receptor pathways upon exposure to GSK.

GO pathway analysis was used to further understand the metabolic and immune response changes in macrophages upon GSK-mediated NOX2 inhibition prior to macrophage activation. Upon GSK treatment, clusters related to metabolic activity, including cholesterol efflux (GO:0033344, fold enrichment = 38.3), cellular modified amino acid metabolism (GO:0006575, fold enrichment = 10.09), and organic acid transport (GO:0015849, fold enrichment = 5.55), displayed increased representation (**Figure 2c**). In addition, clusters associated with wound healing (GO:0042060, fold enrichment = 5.43) and response to oxidative stress (GO:0006979, fold enrichment = 3.81) were also overrepresented, indicating an overall change in macrophage metabolism and immune response activity.

Cellular component-based GO analysis of GSK-treated cells suggested enrichment of genes related to the external side of the plasma membrane (GO:0009897, fold enrichment = 8.91), cell surface (GO:0009986, fold enrichment = 5.79) and side of membrane (GO:0098552, fold enrichment = 5.52), further supporting a change in cell signaling and cell-cell communication upon GSK-based NOX2 inhibition (**Figure 2d**). To further understand the molecular mechanisms of NOX2 inhibition in macrophages, Molecular Function-based GO analysis was performed on the DEGs (**Figure 2e**). Various terms related to transporter activities and transmembrane activities, along with surface signaling receptor activities, were enriched upon GSK treatment. Overall, the effect of GSK alone on naïve macrophages showed a targeted pathway tuning toward an increase in the stress response and cellular communication, but not a gross reprogramming of the cell, as is seen in the response to LPS.

### 3.3 NOX2 inhibition during LPS activation reshaped the macrophage transcriptome and attenuated pro-inflammatory signaling

**Figure 3:**
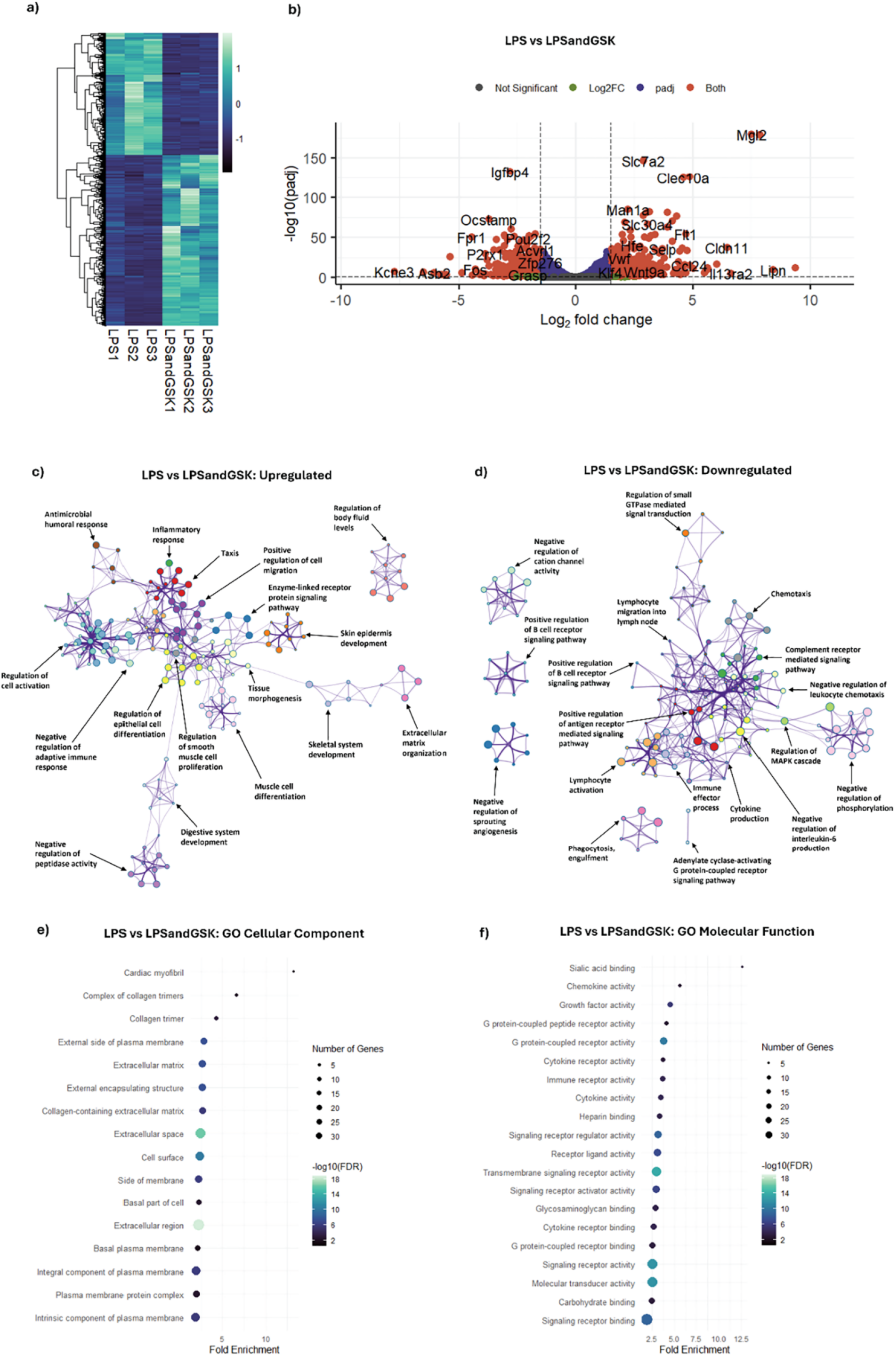
NOX2 inhibition during LPS activation reshapes the macrophage transcriptome and attenuates pro-inflammatory signaling. DeSeq2-based differential expression analysis was performed on RNA-Seq data obtained from synchronized BMDMs exposed to LPS and GSK for 24 h vs. LPS-only treatment for 24 (h) **(a)** Heatmap with unsupervised hierarchical clustering showing the distribution of significant DEGs between LPS and GSK and LPS-only (24 h) treatment. Each column represents one sample, and each row represents one DEG. Z-scores were calculated from normalized gene expression values and were plotted on a scale of -2 to 2. The z-scores are displayed in a dark blue-light green color scheme with dark blue and light green representing low and high expression, respectively. **(b)** Volcano plot indicating the significant upregulated and downregulated DEGs from LPS and GSK treatment vs. control (filter: |Log2 Fold Change| > 1.5 and p-adj < 0.05). Grey dots indicate genes that did not meet significance thresholds. Green dots indicate genes with a fold change above the cutoff that were not statistically significant (p-adj ≥ 0.05). Blue dots represent genes that were statistically significant (p-adj < 0.05) but with a fold change below the cutoff. Red dots represent genes that met both criteria (p-adj < 0.05 and |Log2 Fold Change| > 1.5) and thus were classified as significant DEGs. Representative DEGs are labeled. GO Biological process analysis was performed using Metascape network analysis on the **(c)** upregulated and **(d)** downregulated DEGs from LPS and GSK treatment vs. LPS-only treatment. Significant (p < 0.05) ontology terms were grouped based on similar memberships into color-coded clusters and represented as network plots, with individual nodes representing n enriched terms. Each color-coded cluster is represented by one representative term. Minimum overlap value was set at 3 enriched terms, and minimum enrichment was set as 1.5. **(e)** GO Cellular Component and **(f)** GO Molecular Function-based enrichment analysis was summarized for the top 20 enrichment terms. Categories were selected based on their False Detection Rate (FDR) values and sorted based on their Fold Enrichment, with the point sizes representing the number of genes in each enrichment term and the point color representing the -Log_10_(FDR) value. FDR cutoff was set as 0.05. The complete enrichment results for all three pathway analyses are provided in FASTQ files.

Inhibition of NOX2 has been investigated as a potential therapeutic strategy to modulate excessive ROS production in various inflammatory diseases (10, 43). Given our understanding of the macrophage response to activation and the ability of GSK to tune macrophages to a stress-responsive state, we next sought to determine the effect of NOX2 inhibition on the inflammatory profile of macrophages. To do so, BMDMs were treated with LPS (1 µg/mL) in the presence and absence of GSK (25 µM) for 24 h starting at HPS20. RNA-seq was performed to quantify changes in gene expression. As above, differential expression analysis was conducted using DeSeq2 with a cutoff of |Log_2_FoldChange| > 1.5 and a false discovery adjusted p-value < 0.05 to identify significant DEGs. Of the 15,810 analyzed genes, 1,007 represent significant DEGs as a result of combined and simultaneous LPS + GSK (LPS and GSK) treatment in comparison to LPS-only treated samples. A heatmap with unsupervised hierarchical clustering of the 1,007 significant DEGs shows a distinct gene expression profile from LPS and GSK treatment, indicating that NOX2 inhibition under LPS activation significantly modulates the LPS-mediated pro-inflammatory response (**Figure 3a**). A volcano plot was generated to highlight the upregulated and downregulated significant DEGs (**Figure 3b**). Among the 1,007 significant DEGs, 588 DEGs were upregulated (Log_2_FoldChange > 1.5 and a false discovery adjusted p-value < 0.05) and 419 DEGs were downregulated (Log_2_FoldChange < -1.5 and a false discovery adjusted p-value < 0.05). Among the upregulated DEGs in this comparison, *Mgl2, Clec10a, Klf4, Stat6*, and *Wnt9a* are known to play an important role in modulating the macrophage inflammatory response. The upregulated DEGs also included other immune response-associated genes such as *Slc7a2*, *Flt1*, *Ccl24*, and *Il13ra2*, among others, suggesting that NOX2 inhibition in the presence of LPS activation appears to change the nature of the inflammatory response in macrophages (**Figure 3b**). Among the downregulated DEGs are various immune signaling pathway related genes, including *Igfbp4, Fos, P2rx1*, and *Acvrl1*, which suggests a change in the nature of LPS-mediated macrophage pro-inflammatory response under NOX2 inhibition.

GO pathway enrichment analysis of biological processes indicated that the presence of GSK in LPS-activated macrophages resulted in an increased representation of clusters related to negative regulation of the adaptive immune response (GO:0002820, fold enrichment = 5.39), regulation of cell activation (GO:0050865, fold enrichment = 2.11) and negative regulation of peptidase activity (GO:0010466, fold enrichment = 3.3), suggesting a modulation in macrophage immune activation in the presence of the NOX2 inhibitor (9) (**Figure 3c**). The enrichment of positive regulation of cell migration (GO:0030335, fold enrichment = 2.56) and regulation of epithelial cell proliferation (GO:0050678, fold enrichment = 2.89) clusters indicated a potential role of NOX2 inhibition in facilitating tissue repair and regeneration (44, 45). Network analysis also revealed an enrichment in clusters associated with tissue morphogenesis (GO:0048729, fold enrichment = 2.31) and enrichment of pathways such as the enzyme-linked receptor protein signaling pathway (GO:0007167, fold enrichment =2.71), suggesting alterations in signal transduction in the presence of the NOX2 inhibitor under LPS activation, which might alter macrophage activation and function (46). Among the biological processes enriched in the downregulated DEGs as a result of LPS and GSK treatment in comparison to LPS-only treatment, a central cluster involved pathways related to immune effector processes (GO:0002252, fold enrichment = 2.41), phagocytosis engulfment (GO:0006911, fold enrichment = 7.12), and cytokine production (GO:0001816, fold enrichment = 2.69), including the negative regulation of IL-6 production (GO:0032715, fold enrichment = 6.27) (**Figure 3d**). Enrichment of clusters related to chemotaxis (GO:0006935, fold enrichment = 2.79), negative regulation of leukocyte chemotaxis (GO:0002689, fold enrichment = 10.51), signal transduction pathways including regulation of MAPK cascade (GO:0043408, fold enrichment = 1.89), and regulation of B cell receptor signaling (GO:0050861, fold enrichment = 14.78), among the downregulated DEGs, suggested a broad suppression of signal transduction mechanisms, which might change the inflammatory profile of NOX2 inhibited macrophages (47, 48).

GO pathway analysis of enriched cellular components associated with genes differentially expressed between LPS and GSK treatment and LPS-only treatment also revealed increased representation of clusters related to the extracellular matrix (GO:0031012, fold enrichment = 2.68), including collagen-containing extracellular matrix (GO:0062023, fold enrichment = 2.68), collagen trimer (GO:0005581, fold enrichment = 4.24), and complex of collagen trimers (GO:0098644, fold enrichment = 6.64) (**Figure 3e**). In addition, clusters associated with plasma membrane and other membrane complexes are enriched including cell surface (GO:0009986, fold enrichment = 2.34), external side of plasma membrane (GO:0009897, fold enrichment = 2.82), plasma membrane protein complex (GO:0098797, fold enrichment = 1.91), intrinsic component of plasma membrane (GO:0031226, fold enrichment = 1.86), and integral component of plasma membrane (GO:0005887, fold enrichment = 1.91). These changes could indicate an alteration in the expression or localization of membrane proteins upon NOX2 inhibition (49).

Investigation of molecular functions affected by the presence of the NOX2 inhibitor in LPS-stimulated macrophages revealed a significant enrichment of terms related to immune receptor activity and binding, including G protein-coupled receptor binding (GO:0001664, fold enrichment = 2.6), cytokine receptor activity (GO:0004896, fold enrichment = 3.76), immune receptor activity (GO:0140375, fold enrichment = 3.71), and signaling receptor activity (GO:0038023, fold enrichment = 2.59) (**Figure 3f**). Additionally, clusters associated with chemokine activity (GO:0008009, fold enrichment = 5.67) and growth factor activity (GO:0008083, fold enrichment = 4.55) were enriched as a result of LPS and GSK treatment in comparison to LPS-only treatment. Enrichment of signaling receptor regulator activity (GO:0030545, fold enrichment = 3.23) and transmembrane signaling receptor activity (GO:0004888, fold enrichment = 3.04) indicated changes in signal transduction, while enrichment in heparin binding (GO:0008201, fold enrichment = 3.33), sialic acid binding (GO:0033691, fold enrichment = 12.65), and glycosaminoglycan binding (GO:0005539, fold enrichment = 2.97) indicated changes in extracellular matrix interactions and cell surface dynamics (50–52). In summary, NOX2 inhibition seemed to significantly reshape the transcriptional response of LPS-activated macrophages, attenuating classical pro-inflammatory signaling while enriching pathways related to tissue remodeling, extracellular matrix organization, and immune regulation. These findings indicate that GSK alters not only the magnitude but also the quality of the inflammatory response to LPS.

### 3.4 NOX2 inhibition during LPS activation promoted an anti-inflammatory state via the modulation of oxidative metabolism in macrophages

**Figure 4:**
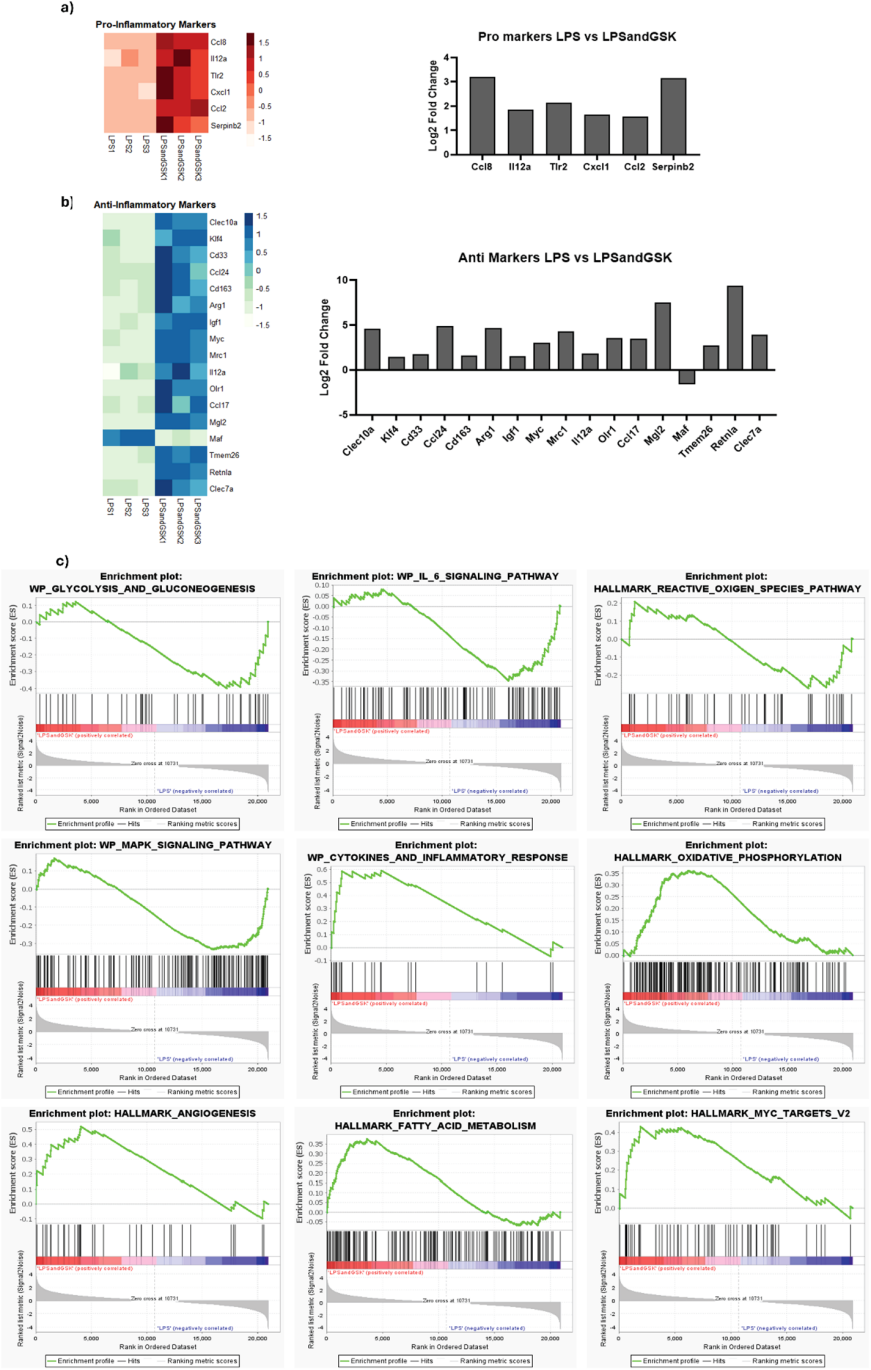
NOX2 inhibition under LPS activation shifts macrophages toward anti-inflammatory, reparative, and oxidative metabolic molecular programs. Heatmap with unsupervised hierarchical clustering (left) and bar plot showing the Log_2_FoldChange in levels (right) of **(a)** pro-inflammatory and **(b)** anti-inflammatory markers in LPS and GSK treated samples in comparison to LPS-only-treated samples. **(c)** GSEA was performed on ranked differential gene expression data to identify pathways enriched in LPS and GSK and LPS-only conditions. Pathways with p-value < 0.05 and False Discover Rate (FDR) < 0.25 (software default setting) were considered significant and were used for the analysis. Each panel shows an enrichment plot, where the green line represents the enrichment score (ES) for each gene set as the analysis walks down the ranked gene list in that gene set. Vertical black bars indicate the position of individual genes from the gene set within the ranked list. Pathways with positive enrichment scores are upregulated in LPS and GSK relative to LPS-only, while those with negative scores are downregulated. Representative pathways enriched in the LPS-only condition included glycolysis, IL-6 signaling, and ROS production, whereas pathways enriched in the LPS and GSK condition included oxidative phosphorylation, fatty acid metabolism, and angiogenesis. The gray curve underneath the enrichment plot represents the Signal2Noise metric for each gene, with a higher value indicating stronger correlation with LPS and GSK (left) condition. and lower value correlation with LPS (right) condition. Statistical significance was determined using 1,000 label gene set-based permutations.

Having established broad transcriptomic reprogramming upon the addition of LPS and GSK in macrophages, our next goal was to determine how these changes translate into shifts in specific pro- and anti-inflammatory marker genes and associated metabolic pathways. Therefore, we transitioned our focus to an analysis of signature cytokines, chemokines, and metabolic regulators that defined the inflammatory phenotype of macrophages under LPS and GSK co-treatment (**Figures 4a, b**). Among the significant DEGs between LPS and LPS and GSK treated macrophages, expression of pro-inflammatory markers *Ccl8, Il12a, Tlr2, Cxcl1, Ccl2*, and *Serpinb2* were surprisingly significantly increased as a result of LPS and GSK treatment compared to LPS-only treatment (**Figure 4a**). Chemokines *Ccl8, Cxcl1*, and *Ccl2* play a key modulatory role in macrophage inflammatory response (53). The upregulation of *Il12a* and *Tlr2* further indicates ongoing pro-inflammatory signaling even in the presence of the NOX2 inhibitor GSK.

Contrary to the further upregulation of pro-inflammatory markers, the co-treatment with LPS and GSK also resulted in increased expression of anti-inflammatory markers (**Figure 4b**). Included in this list are *Clec10a, Klf4, Ccl24*, and *Mgl2*, anti-inflammatory markers that contribute towards tissue repair and alternative activation in macrophages (**Figure 4b**) (54–56). In addition, *Cd163*, *Arg1*, *Mrc1* (also known as *CD206*), *Igf1*, and *Ccl17*, characteristic markers of anti-inflammatory activation in macrophages, were also upregulated (57–61). Their gene products, Myc, Olr1, Tmem26, and Retnla, affect various aspects of macrophage metabolism, oxidative stress response, and signaling involved in the alternative activation of immune response (28, 62, 63). The increased expression of these anti-inflammatory markers as a result of LPS and GSK treatment in comparison to LPS-only treatment highlights the complex interplay between pro- and anti-inflammatory pathways and indicates that NOX2 inhibition modulates the inflammatory pathways leading to an anti-inflammatory state via a complex mechanism, likely only partially related to the classical anti-inflammatory pathways.

To further investigate the transition of macrophages into a more anti-inflammatory phenotype upon NOX2 inhibition under LPS activation, Gene Set Enrichment Analysis (GSEA) was performed on the transcriptomic data from BMDMs treated with LPS and GSK vs. LPS-only treatment. Using GSEA, genes are ranked based on their differential expression (including Log_2_FoldChange value and adjusted p-value) between two biological conditions, enabling the calculation of an enrichment score for each gene set by assessing the overrepresentation of its constituent genes at the top or bottom of the ranked list (64). GSEA of LPS and GSK RNA-Seq data in comparison to LPS-only RNA-Seq data revealed significant differences in metabolism and signaling pathways due to LPS and GSK treatment (**Figure 4c**). This is relevant as, beyond cytokine and chemokine production, the polarization of macrophages into pro- and anti-inflammatory phenotypes is also driven via the generation of distinct metabolic profiles (33). LPS-only treatment resulted in increased enrichment of several pathways, including glycolysis and gluconeogenesis, IL-6 signaling, ROS production, and MAPK signaling, in comparison to LPS and GSK treatment (**Figure 4c**). This change is logical, as pro-inflammatory activated macrophages rely heavily on glycolysis for rapid ATP production that supports the biosynthesis of various intermediates required for sustaining the inflammatory response (34). In contrast, LPS and GSK treatment resulted in significantly increased enrichment of oxidative phosphorylation, angiogenesis, and fatty acid metabolism pathways (**Figure 4c**). Macrophages under anti-inflammatory activation maintain an intact Krebs cycle and rely heavily on oxidative phosphorylation and fatty acid oxidation for energy catabolism (33, 65–67). The enrichment of these pathways, along with angiogenesis-associated pathways, supports a view of the reprogramming of NOX2-inhibited macrophages towards anti-inflammatory functions, even in the presence of LPS, via the modulation of metabolic states (65, 67).

### 3.5 NOX2 inhibition reduces ROS production and shifts cytokine secretion from pro-inflammatory to anti-inflammatory profiles in LPS-activated macrophages *in vitro*

**Figure 5:**
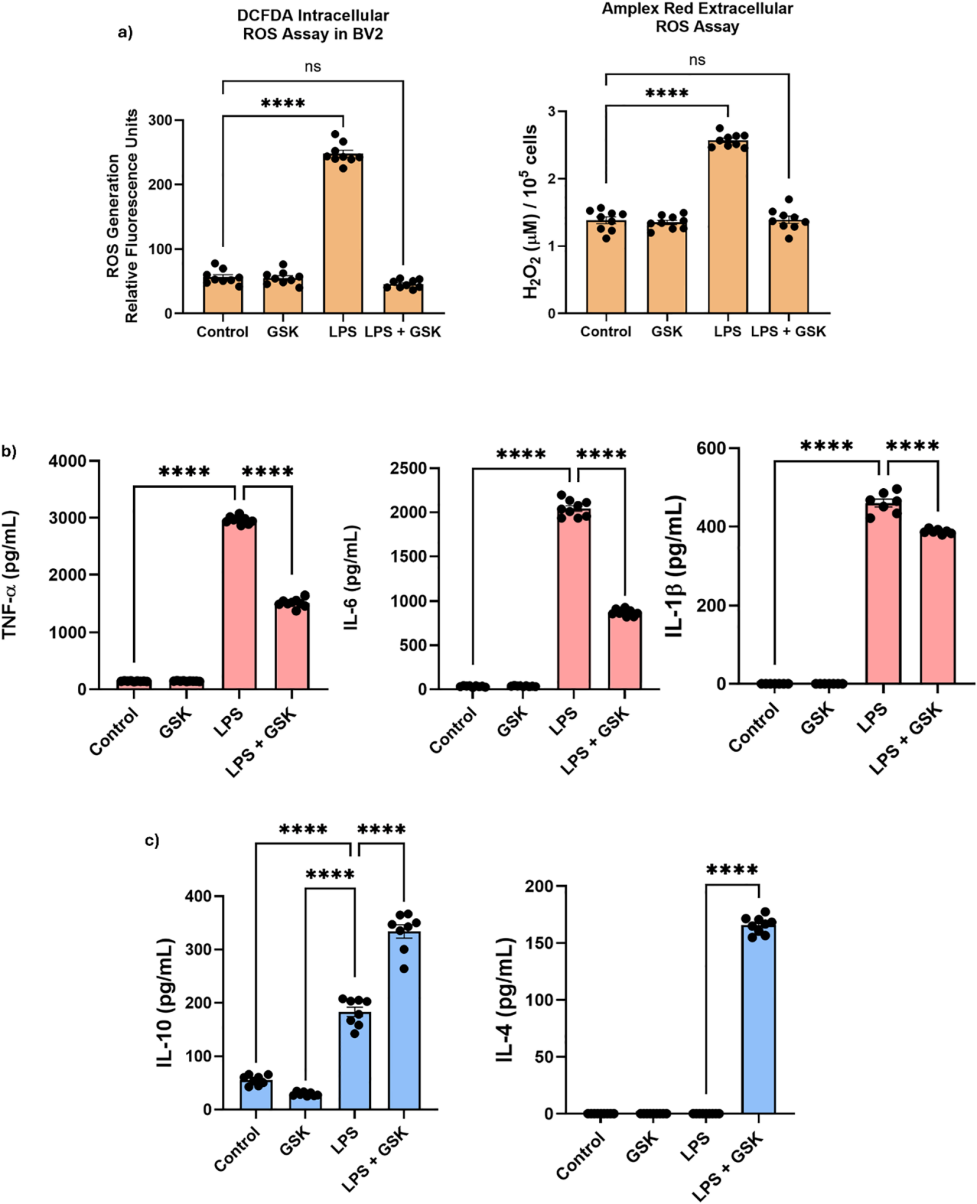
Functional validation of NOX2 inhibition shows reduced ROS and inflammatory cytokine release in LPS-activated macrophages. **(a)** Intracellular and extracellular ROS levels in LPS-activated macrophages with or without GSK. Levels of **(b)** pro-inflammatory cytokine TNF-α, IL-6, and IL-1β, and **(c)** anti-inflammatory cytokines IL4 and IL-10 measured in the media supernatant of LPS-activated macrophages with or without GSK. Data shown as mean ± SEM and were analyzed using a single-factor ANOVA test. For all statistical analyses, ns denotes non-significant and **** denotes p < 0.0001.

Based on the changes in inflammatory markers and metabolic pathways, we hypothesized that macrophages treated with GSK in the presence of LPS would lead to a decrease in ROS and pro-inflammatory cytokines in the macrophages themselves. Hence, we next measured the levels of intracellular and extracellular ROS, and select pro- and anti-inflammatory cytokines, in BMDMs to further validate our view of the effect of the molecular reprogramming of macrophages due to NOX2 inhibition under LPS activation *in vitro*. ROS levels were assessed in BMDMs using a DCFDA assay (to measure intracellular ROS) and an Amplex Red assay (to measure extracellular ROS). As before, cells were activated using LPS (1 μg/mL) and were incubated with (25 µM) or without GSK, while untreated macrophages served as a control. LPS-only treated cells had significantly increased intracellular ROS production compared to the untreated control cells, as expected (**Figure 5a**). This is consistent with previous studies in mouse macrophages where LPS, a potent activator of TLR4, induces NOX2-dependent ROS production (9). In addition, and in concordance with our RNA-seq data, treatment with GSK did not affect basal ROS levels compared to the control, suggesting that NOX2 inhibition does not affect basal ROS production in inactivated macrophages (9). Finally, co-treatment of LPS and GSK significantly reduced ROS levels to control levels (**Figure 5a**). This result further validates previous observations in macrophages where NOX2 inhibition significantly reduced ROS production (12, 68). Furthermore, extracellular ROS levels displayed similar trends with intracellular ROS levels, with LPS activation significantly increasing ROS production as compared to the untreated control, and co-treatment of LPS and GSK resulted in ROS levels comparable to the untreated control (**Figure 5a**).

The aforementioned DeSeq2 and GSEA results indicated an increase in anti-inflammatory marker expression due to LPS and GSK treatment in comparison to LPS-only treatment. To confirm this *in vitro*, and to understand the effect of NOX2 inhibition on the inflammatory response of LPS-activated macrophages, the expression of select pro-inflammatory markers (TNF-α, IL-6, and IL-1β) and anti-inflammatory markers (IL-10 and IL-4) were measured. Cells were stimulated using LPS with or without GSK for 24 h, with untreated cells serving as control. ELISA analysis of cytokine expression levels revealed a significant increase in the levels of pro-inflammatory cytokines, TNF-α, IL-6, and IL-1β, upon LPS activation (**Figure 5b**). This increase aligns with previously observed trends wherein LPS activation resulted in a pro-inflammatory response in macrophages through the activation of TLR4-mediated NF-κB, promoting the secretion of various pro-inflammatory markers (69). The treatment of GSK alone did not alter the expression of these pro-inflammatory cytokines, with levels staying comparable to that of the untreated control. The co-treatment of LPS and GSK resulted in a significant decrease in the expression of all three pro-inflammatory cytokines compared with LPS-only incubation. TNF-α (~50% reduction in levels compared to LPS-treated samples) and IL-6 (~50% reduction in levels compared to LPS-treated samples) displayed a greater decrease in expression upon co-treatment of LPS and GSK compared to IL-1β (~13% reduction in levels compared to LPS-treated samples), which displayed a slight yet significant decrease. These results further support the hypothesis that NOX2-generated ROS contributes to the amplification of pro-inflammatory cytokines (e.g., TNF-α, IL-6, and IL-1β) during an inflammatory response in macrophages, and that NOX2 inhibition dampens the inflammatory cascade initiated by LPS activation, though not completely (as was predicted by our RNA-seq analysis) (9, 70, 71).

In contrast to pro-inflammatory cytokines, LPS stimulation had no effect on the expression of the anti-inflammatory cytokine IL-4 (**Figure 5c**). While GSK treatment alone did not alter IL-4 expression levels, co-treatment with LPS and GSK displayed a significant increase in IL-4 production (~150% increase in levels compared to LPS-treated samples). Similar to IL-4, GSK treatment alone did not alter the levels of the anti-inflammatory cytokine IL-10 (**Figure 5c**). While LPS treatment resulted in a moderate increase in IL-10 levels, the co-treatment of LPS and GSK significantly enhanced IL-10 production (~82% increase in levels compared to LPS-treated samples). This increase in IL-4 and IL-10 secretion in macrophages suggests that NOX2 inhibition not only reduces pro-inflammatory cytokine production, but also supports a shift towards an anti-inflammatory response, even under LPS activation (12, 72).

## 4 Discussion

Inhibition of ROS has emerged as a potential therapeutic strategy to modulate inflammation and oxidative stress in various diseases (68). NOX2 inhibitors, such as GSK2795039, have demonstrated potential in reducing oxidative stress and dampening inflammation in preclinical models (11, 12). NOX2 inhibition has also been associated with a shift in macrophage polarization from a pro-inflammatory phenotype towards an anti-inflammatory phenotype (12, 73). This polarization shift is crucial in diseases where chronic inflammation impedes tissue repair and regeneration (74). By promoting an anti-inflammatory phenotype in macrophages, NOX2 inhibition may enhance tissue repair processes and thus, support the resolution of inflammation (73). While the effect of NOX2 inhibition has been studied in various models at the cellular level, gaps remain in our understanding of how NOX2 inhibition affects macrophages at the molecular level, leading to a mechanistic change in their metabolic and functional responses. In this study, we investigated the effects of NOX2 inhibition at the transcriptional level in BMDMs as a result of adding GSK. Using this approach, we identified differentially expressed genes (DEGs) as a result of GSK treatment after LPS stimulation. Through comprehensive gene ontology (GO) pathway analyses of these DEGs, we were able to explore the molecular reprogramming induced by NOX2 inhibition and its broader implications for macrophage metabolism, immune function, and cellular communication. We acknowledge that our study employs high concentrations of both LPS and GSK compared to some previous reports. These concentrations were selected based on prior studies to ensure robust NOX2 inflammatory activation and inhibition, respectively, without inducing cytotoxicity, as confirmed by viability assays (**Supplementary Figure S2**).

The transcriptional changes observed in BMDMs upon LPS activation supported the intense reprogramming the cells undergo in response to a pro-inflammatory stimulus. Among the upregulated DEGs were many genes that mediate and amplify the inflammatory response in macrophages, including *Il6*, *Il1a*, *Il12b*, *Cd274*, *Cxcl9*, and *Lcn2* (75). While *Il6* and *Il1a* are key cytokines that promote inflammation and immune responses in macrophages, *Il12b* and *Cd274* are involved in the activation and modulation of T cell responses (75–78). Upregulated *Rsad2*, *Ifit1*, *Ifih1*, and *Mx1* fall under the interferon-stimulated gene (ISG) response, indicating an increase in the innate immunity response and antiviral defense mechanism in LPS-treated macrophages (79). In total, these findings are consistent with previous studies that have demonstrated the broad spectrum of immune responses resulting from LPS activation (1, 3, 33–36).

Concordantly, the downregulation we noted of genes associated with anti-inflammatory processes, such as *Stab1*, *Cd163*, *Clec10a*, and *Pros1*, further emphasized the transition towards a predominantly pro-inflammatory phenotype upon LPS activation. *Stab1* and *Cd163* act as scavenger receptors and, along with *Clec10a*, are involved in anti-inflammatory processes and the clearance of cellular debris (80). PROS1 serves as a ligand for TAM receptors, and its decreased expression results in the increased production of pro-inflammatory cytokines TNF-α and CCL3 (81). Downregulation of *Stab1*, *Cd163*, *Clec10a*, and *Pros1* further suggests a suppression of anti-inflammatory pathways in favor of a pro-inflammatory phenotype.

These transcriptional changes are further supported by the Gene Ontology (GO) analyses, which demonstrated the global downregulation of pathways related to cell cycle progression and DNA replication, suggesting that macrophages enter a more differentiated and functionally active state rather than focusing on proliferation upon LPS activation. The enrichment of pathways associated with cytokine production, immune receptor activity, and signal transduction additionally supported the shift of macrophages towards an activated, pro-inflammatory phenotype upon exposure to LPS (5).

Prior to examining the combined effects of LPS and GSK, the impact of GSK alone was assessed to serve as a reference point. As expected, GSK treatment in naïve macrophages led to only modest transcriptional changes (42 significant DEGs out of 17,763 analyzed genes), with limited numbers of significant DEGs primarily linked to metabolism, oxidative stress, and membrane signaling. Among the upregulated genes, *Cox6a2* (a mitochondrial respiratory chain subunit) and *Dio2* (involved in thyroid hormone metabolism) suggest alterations in mitochondrial and metabolic activity, while *Slc7a11* (a cystine/glutamate antiporter) and *Trib3* (a stress response regulator) highlight changes in redox balance and stress signaling (82–84). Conversely, downregulation of *Abca1* and *Abcg1* (key cholesterol efflux transporters) points to altered lipid handling, while reduced expression of *Clec4a* and *Ccr3* suggests a change in immune receptor signaling (85–87). These findings suggest that acute NOX2 inhibition in naïve macrophages does not trigger large-scale reprogramming, but rather fine-tunes pathways related to the stress response, metabolism, redox homeostasis, and cell surface signaling.

In contrast to the minor change in transcriptional programming affected by GSK treatment, the analysis of the co-treatment of LPS and GSK demonstrated a considerably reshaped macrophage transcriptomic profile. The combined treatment of LPS and GSK resulted in a distinct shift in the macrophage inflammatory response and metabolism. The upregulation of genes, such as *Mgl2*, *Clec10a*, *Klf4*, and *Wnt9a*, in the LPS and GSK treated macrophages suggested a transition towards an anti-inflammatory or reparative phenotype (88). *Mgl2* is a C-type lectin receptor that is expressed on the surface of macrophages and plays an important role in recognizing terminal GalNAc residues on pathogens and apoptotic cells (89). *Mgl2* triggers IL-10 production by macrophages and is induced in macrophages and microglia with anti-inflammatory phenotypes, thus participating in the resolution of inflammation (90). The upregulation of *Mgl2* in response to NOX2 inhibition under LPS activation suggests enhanced pathogen recognition, phagocytosis, and a shift towards a more anti-inflammatory macrophage phenotype. *Clec10a*, encoding for an anti-inflammatory marker, is another C-type lectin domain family member that is involved in antigen uptake, modulation of immune response, and maintenance of tissue homeostasis (91). The increased expression of *Clec10a* indicated a potential shift towards a less inflammatory cellular phenotype, suggesting improved antigen processing and immune surveillance (91). The gene product of *Klf4* interacts with the gene product of *Stat6* to induce an anti-inflammatory macrophage phenotype, and *Wnt9a* is part of the Wnt signaling pathway, which plays a role in regulating inflammation and promoting tissue repair (92). In total, the upregulation of these genes suggests that NOX2 inhibition might enhance signaling pathways that contribute to a more anti-inflammatory response, even in the presence of LPS.

In addition to these genes, NOX2 inhibition in LPS-treated macrophages resulted in upregulation of other immune response-associated genes beyond those that regulate inflammation, including *Slc7a2*, *Flt1*, *Ccl24*, and *Il13ra2*. *Slc7a2* encodes a cationic amino acid transporter important for arginine transport (93). With arginine being the substrate for nitric oxide synthase, the upregulation of this gene could enhance macrophage function through the modulation of nitric oxide production (94). *Flt1* is involved in the regulation of angiogenesis and cell migration (95). Increased expression of *Flt1* suggests a role of NOX2 inhibition in a transition from inflammation to tissue repair and regeneration (96). Increased expression of *Ccl24*, a chemokine primarily involved in the recruitment of other immune cells, including eosinophils, to sites of inflammation, might indicate a shift towards an amplified immune response with a shift towards tissue repair and resolution of inflammation (36). Similarly, Il13ra2 is a decoy receptor that influences the effects of the cytokine IL-13, which plays a crucial role in inflammation (97). Increased expression of *Il13ra2* may indicate a varied macrophage inflammatory response resulting from the modulation of IL-13 levels (98). The increased expression of all these genes further supported a change in the macrophage inflammatory profile upon NOX2 inhibition under LPS activation.

Not only were many genes that reduce inflammation upregulated upon NOX2 inhibition, but many pro-inflammatory genes and pathways were downregulated, including *Igfbp4*, *Fos*, and *P2rx1*, suggesting suppression of the inflammatory cascade in LPS and GSK treated macrophages. Among these, Igfbp4 is a binding protein that regulates the levels of insulin-like growth factors (IGFs) in macrophages (99). Due to IGFs crucial role in cell growth, migration, and macrophage polarization towards an anti-inflammatory phenotype, a decreased expression of *Igfbp4* may indicate an enhanced availability of IGFs resulting in a shift towards tissue repair and resolution of inflammation (99). Fos, a component of the activator protein-1 (AP-1) complex, regulates the expression of various genes involved in inflammation, including cytokines and chemokines, such as TNF-α, IL-6, and IL-1β (100). *Fos*, through its role in AP-1, also influences the expression of genes involved in oxidative stress and ROS production (101). Reduced *Fos* expression suggests a shift towards an anti-inflammatory phenotype with reduced inflammation levels (4). *P2rx1* is involved in ATP-mediated signaling, which contributes to the production of TNF-α and IL-1β, and NLRP2 inflammasome activation (102, 103). Decreased *P2rx1* expression suggests a more anti-inflammatory environment resulting from limited pro-inflammatory activation (103). Along with the enrichment of pathways associated with tissue repair, immune regulation, and signal transduction, downregulation of these genes underscores the transition of macrophages into an anti-inflammatory phenotype upon NOX2 inhibition under pro-inflammatory activation. Notably, the use of pharmacological inhibition via GSK in fully differentiated BMDMs contrasts with prior studies using NOX2 knockout models that have indicated that NOX2 derived ROS might restrain pro-inflammatory signaling and help promote immunological tolerance (104–107). While valuable, these knockout models may elicit developmental compensation and broader redox imbalances (108). This acute, context-specific inhibition under LPS activation likely reveals stimulus dependent roles of NOX2, highlighting that in certain pro-inflammatory settings, NOX2 activity amplifies rather than restrain inflammatory responses (12, 73, 108–111).

The shift in transcriptional phenotype in macrophages upon GSK treatment after LPS exposure was further demonstrated by increased levels of various anti-inflammatory markers, such as *Arg1, Mrc1, Cd163*, and *Ccl17*, in LPS and GSK treated macrophages. *Cd163* is a scavenger receptor involved in the clearance of hemoglobin-haptoglobin complexes and is a strong marker of anti-inflammatory macrophage activation, reported to induce the secretion of anti-inflammatory cytokines (57). Arginase 1 (coded by *Arg1*) is an enzyme that competes with iNOS for their common substrate, L-arginine, and is a classic marker of an anti-inflammatory phenotype through its involvement in establishing tissue repair and homeostasis (58). *Mrc1* (*Cd206*) is another well-established marker of anti-inflammatory activated macrophages that recognizes mannose residues on the surface of pathogens and facilitates phagocytosis (59). *Ccl17* is involved in the suppression of immune responses and promotes macrophage tolerance by recruiting regulatory T cells (61). Interestingly, recent evidence has shown that NOX2 inhibition by GSK2795039 (GSK) can enhance macrophage efferocytosis via the MertK/PI3K/AKT pathway, contributing to plaque stabilization *in vivo (*111). The upregulation of *Cd163, Clec10a, Klf4*, alongside increased expression of IL-10 and IL-4 as seen in this work suggest that GSK treatment may promote a reparative macrophage phenotype supportive of efferocytosis (105–107, 111).

Conversely, the increased expression of certain pro-inflammatory markers, including *Ccl8, Il12a, Tlr2*, and *Serpinb2*, in LPS and GSK treated macrophages highlights the complex interplay between pro- and anti-inflammatory signaling pathways and the range of inflammatory responses that fall under the control of NOX2 (112). It also suggests that while NOX2 inhibition under LPS activation dampened some aspects of the pro-inflammatory immune response, the cells continue to display pro-inflammatory signaling outside the range of GSK-specific NOX2 inhibition.

To help explain decreased inflammatory phenotypes in the presence of an increase in a limited set of inflammatory markers, our Gene Set Enrichment Analysis (GSEA) of these DEGs also showed a reduced enrichment of glycolysis, IL-6 signaling, ROS production, and MAPK signaling pathways – key metabolic and inflammatory pathways in pro-inflammatory macrophages (75). Pro-inflammatory activated macrophages rely heavily on glycolysis for rapid ATP production, which supports the biosynthesis of various intermediates required for sustaining the inflammatory response (34). The reduced enrichment of glycolysis-associated pathways in LPS and GSK samples reflects a change in the functional capabilities of NOX2-inhibited macrophages under LPS-only activation (34, 113). In addition, the reduced enrichment of IL-6, ROS, and MAPK pathways in LPS and GSK treated cells complemented this change in the metabolic state, indicating a shift away from the pro-inflammatory phenotype, and potentially reflecting the influence of NOX2 inhibition in tempering the inflammatory response (33, 114). Macrophages under anti-inflammatory activation maintain an intact Krebs cycle and rely heavily on oxidative phosphorylation and fatty acid oxidation for energy generation (33, 65–67). In concordance with this, we noted increased enrichment of oxidative phosphorylation, fatty acid metabolism, and angiogenesis pathways in LPS and GSK treated cells (65).

Finally, the effect of the molecular reprogramming predicted by our transcriptomic results was confirmed by *in vitro* cell-based studies, highlighting the critical role of NOX2 in modulating the inflammatory response in macrophages. The significant reduction in both intracellular and extracellular ROS levels in LPS-activated macrophages treated with GSK supports the central role NOX2 plays in ROS production during the macrophage inflammatory response (74). In addition, the fact that GSK alone did not alter basal ROS levels suggests that NOX2 is primarily responsible for LPS-induced pro-inflammatory ROS burst, rather than maintaining baseline ROS production (74). The reduction in the expression of pro-inflammatory genes in LPS and GSK treatment is mirrored by the significant decrease in the production of the pro-inflammatory cytokines, TNF-α, IL-6, and IL-1β. Importantly, the concomitant increase in the levels of anti-inflammatory cytokines IL-4 and IL-10 as a result of LPS and GSK treatment further consolidates the shift into a more anti-inflammatory-like macrophage response.

Taken together, our findings build on the classical understanding of LPS as a pro-inflammatory stimulus by demonstrating that NOX2-derived ROS not only amplify TLR4-driven inflammatory response but also modulate the signaling environment. Pharmacological inhibition of NOX2 attenuates these effects and permits the emergence of anti-inflammatory and reparative gene expression programs.

## 5 Conclusions

GSK-mediated NOX2 inhibition significantly impacts the transcriptome and inflammatory profile of LPS-treated macrophages. NOX2 inhibition leads to significant alterations in LPS-stimulated macrophage metabolism and immune response, shifting them towards an anti-inflammatory and reparative phenotype, even under pro-inflammatory conditions. The upregulation of genes involved in the antioxidant response and immune modulation highlights a reprogramming of macrophages that might enhance their ability to cope with oxidative stress while tempering excessive inflammatory signaling. Conversely, the downregulation of genes associated with lipid metabolism, cell migration, and immune activation suggests that NOX2 inhibition not only reduces ROS production but also mitigates macrophage-driven inflammation. These results underline the potential of targeting NOX2 in diseases characterized by chronic inflammation. It should be noted that the current study used relatively high (but not out of range of the literature) LPS and GSK concentrations, and thus, the results may be specific to BMDMs. Further research is warranted to explore the broader implications of NOX2 inhibition on macrophage function and its potential role in treating inflammatory diseases.

## Data Availability

The original contributions presented in the study are publicly available. This data can be found here: https://data.mendeley.com/datasets/ggndrstwd2/1.
