# Supplementary material for: Integrated transcriptomic and functional analyses reveal that NOX2 inhibition rewires the inflammatory landscape of macrophages
Source: Front Immunol. 2026 Feb 18;17:1731888. doi: 10.3389/fimmu.2026.1731888 (PMC12956517; doi:10.3389/fimmu.2026.1731888)
Supplement: Supplementary file 1 [file DataSheet1.pdf]

Supplemental Material

Integrated Transcriptomic and Functional Analyses Reveal that NOX2 Inhibition Rewires the Inflammatory Landscape of Macrophages

Iswarya Muthukumarasamy[1,2], Sharleen M. Buel[2,3,4], Jennifer M. Hurley[2,3,4*], and Jonathan S. Dordick[1,2,3,4*]

[1]Department of Chemical and Biological Engineering
[2]Center for Biotechnology and Interdisciplinary Studies
[3]Department of Biological Sciences

Rensselaer Polytechnic Institute
Troy, NY 12180

[4]Rensselaer-Mount Sinai Center for Engineering and Precision Medicine
619 W. 54th Street
New York, NY 10019

a)

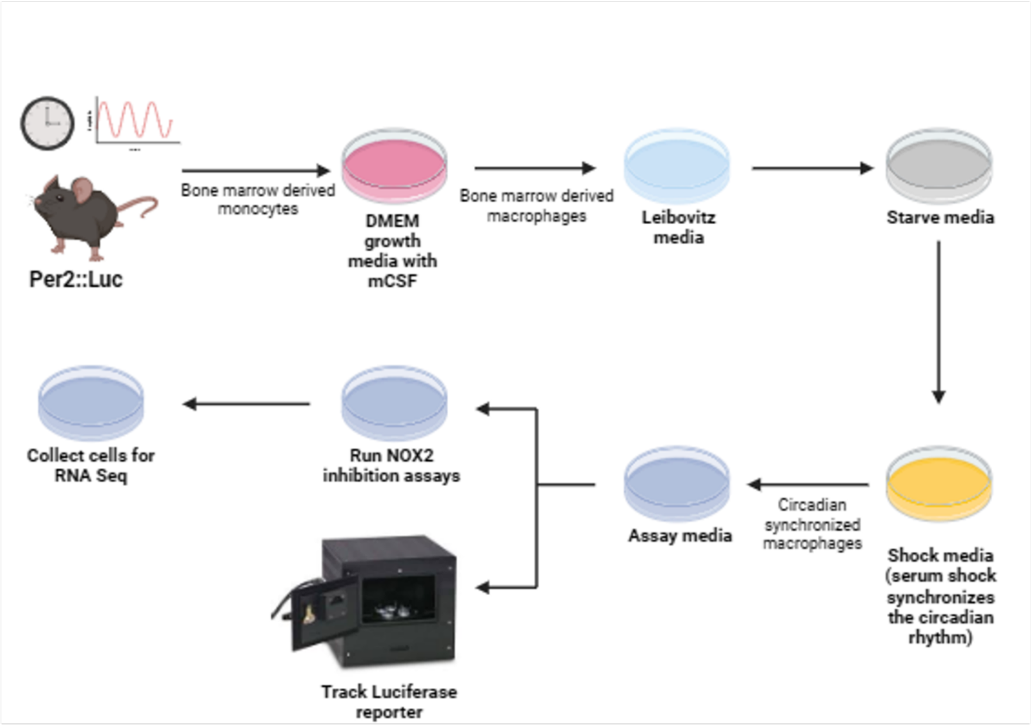

b)

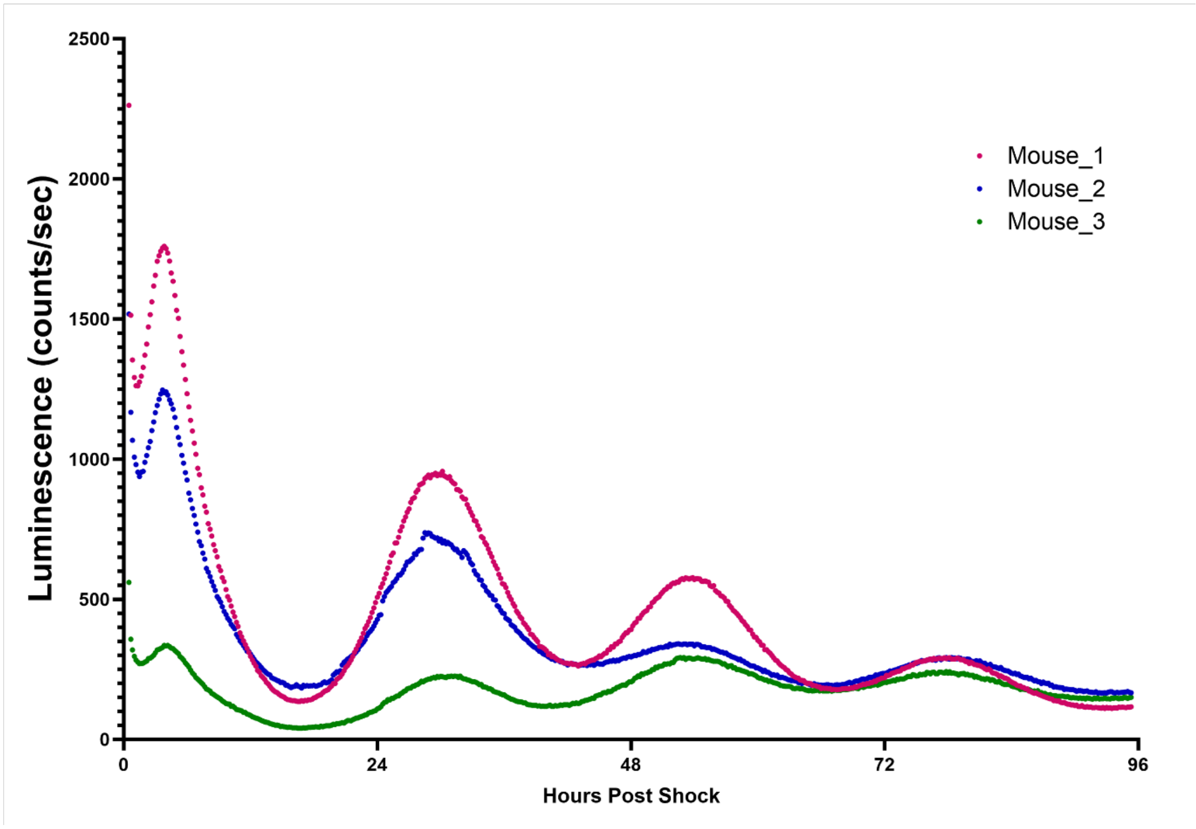

Figure S1: Serum shock synchronization protocol. a) Monocytes were obtained from the femur of mice grown in 12 h dark and 12 h light standard growth condition. Monocytes were differentiated into macrophages using M-CSF. The

resultant cells were serum shock synchronized prior to the treatment of the NOX2 inhibitor, GSK2795039, with and without LPS (samples with no treatment was used as Control). Circadian synchronization in the samples was validated by measuring luciferase levels in a LumiCycle. Differential gene expression analysis was performed on RNA-seq data obtained from the resultant BMDMs treated with GSK with and without LPS for 24 h, and was compared with control samples with no inhibitor. **b)** Luciferase traces of the control cell samples obtained from the three biological replicate mice post serum shock, confirming synchronization of the circadian clock at the level of the PERIOD2 promoter.

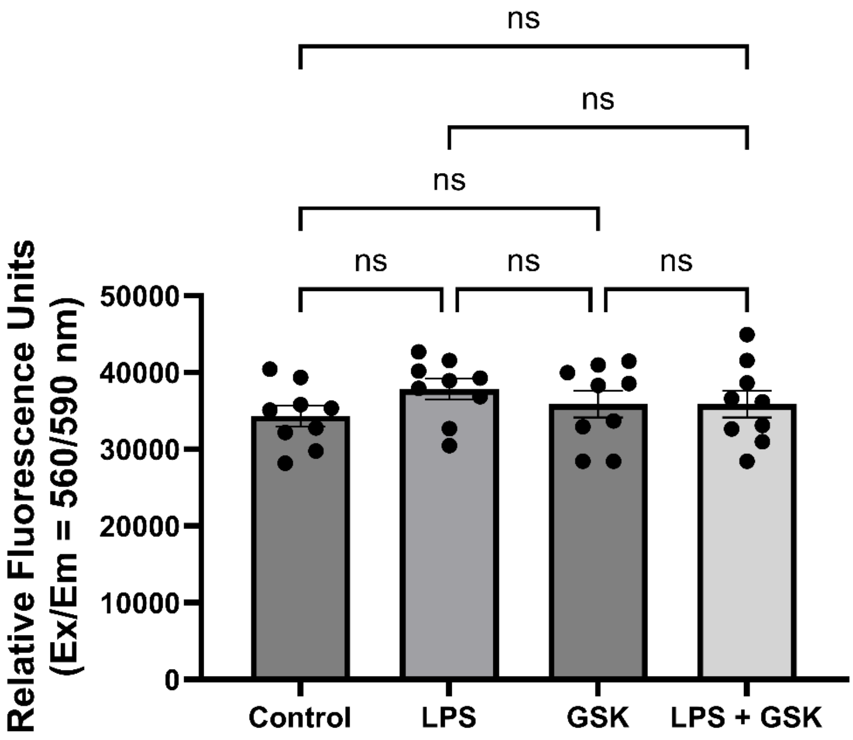

Figure S2: Cell viability results for different treatment conditions. BMDMs were treated for 24 hours with either vehicle control (Control), LPS (1 μg/mL), GSK (25 μM) or LPS (1 μg/mL) + GSK (25 μM). Cell viability was assessed using the PrestoBlue Cell Viability Assay (fluorescence Ex/Em = 560/590 nm). Results are shown as Relative Fluorescence Units (RFU) with each bar representing the mean ± SEM and analyzed using single-factor ANOVA test. For all statistical analyses, 'ns' denotes non-significant. The results demonstrate that neither LPS nor GSK, alone or in combination, induced detectable cytotoxicity under the experimental conditions.
